# Testing Women With Endometrial Cancer for Lynch Syndrome: Should We Test All?

**DOI:** 10.6004/jadpro.2013.4.5.4

**Published:** 2013-09-01

**Authors:** Jun Ma, Nancy Ledbetter, Lyn Glenn

**Affiliations:** From Providence Cancer Center, Providence Portland Medical Center, Portland, Oregon

## Abstract

Women with Lynch syndrome (LS) are at equal or higher risk for gynecologic cancers compared with their risk for colorectal cancer (CRC). Endometrial cancer (EC) often precedes CRC as patients’ sentinel malignancy. Identifying these patients is believed to reduce their substantial risk for synchronous and metachronous tumors and has profound implications for reducing cancer-related morbidity and mortality in other family members. Routine screening of patients with CRC for LS has become increasingly common, but routine screening for LS in women with EC is rarely performed. Current screening guidelines for identifying LS in women with EC vary but rely heavily on patient age and personal/family history, with or without incorporation of tumor pathology. Because each of these strategies misses a significant proportion of women with LS, more inclusive screening strategies that make good economic and clinical sense are needed. In recent years, emerging medicoeconomic evidence supports the fact that screening EC patients for LS may be cost-effective. Implementation of such a strategy requires multidisciplinary collaboration and partnership.

Lynch syndrome (LS), also known as hereditary nonpolyposis colorectal cancer, is one of the most common autosomal-dominant cancer syndromes. It is estimated to affect between 1 in 500 and 1 in 1000 individuals. Lynch syndrome increases an individual’s risks for developing cancers of the colon, endometrium, ovary, upper gastrointestinal tract, small bowel, hepatobiliary system, pancreas, ureter/renal pelvis, and brain (Beamer et al., 2012; Lynch & Casey, 2007; Umar et al., 2004). The frequency of LS among endometrial cancer (EC) patients, reported to be 1.8% to 3%, is believed to be similar to the reported approximately 3% in colorectal cancer (CRC) patients (Hampel et al., 2005a, 2006). The lifetime risk of EC in women with LS is estimated to be from 30% to over 60%, which may be comparable to or may surpass their lifetime risk of CRC. The average age at diagnosis is 47 to 55 (Hampel et al., 2005b).

Endometrial cancer in women with LS is more often associated with MSH2 and MSH6 mutations than with mutations in the MLH1 or PMS2 genes. Women carrying an MSH6 mutation have an estimated lifetime EC risk of 71% by age 70 (Hendriks et al., 2004). The average woman has a 2% to 3% lifetime risk of endometrial cancer (sporadic ED) as compared to the significantly increased risk in women with LS. The average age at diagnosis in the mid-60s. The lifetime risk for developing ovarian cancer (OC) is 12% in women carrying a deleterious mutation. Synchronous cancers in both the ovary and endometrium have been reported in over 20% of patients with LS (Lynch & Casey, 2007).

A substantial number of women with LS first present with a sentinel gynecologic cancer (EC in particular), and these patients are at increased risk for synchronous and metachronous tumors, especially CRC (Lynch & Casey, 2007; Meyer, Broaddus, & Lu, 2009). The median latency period between EC and colon cancer is reported to be 11 years; the median latency period is 5.5 years for patients with a sentinel ovarian cancer (Lu et al., 2005). Timely identification of these women in a cost-effective manner has important clinical and financial implications: It allows patients and their family members to receive appropriate surveillance and have an opportunity to discuss risk-reducing strategies that may reduce the morbidity and mortality associated with LS-related cancers (Longacre & Folkins, 2011).

Routine screening of CRC patients for LS is becoming increasingly common (Beamer et al., 2012; Evaluation of Genomic Applications in Practice and Prevention [EGAPP] Working Group, 2009), but routine screening for LS in women with EC is rarely performed. However, emerging medicoeconomic evidence supports the fact that screening EC patients for LS may be cost-effective and calls for gynecologic oncologists and pathologists to actively participate in studies that evaluate the efficacy of routine screening of EC patients for LS.

## Molecular Background

Lynch syndrome is caused by a germline mutation in one of the four mismatch repair (MMR) genes—MSH2, MSH6, MLH1, and PMS2—or less often by a germline mutation in the epithelial cell adhesion molecule (EPCAM) gene. When functioning properly, MMR genes help prevent cancer by recognizing and repairing mistakes that arise during DNA replication. Mutations in the MMR genes lead to uncontrolled cell growth and possible malignant transformation of cells.

Microsatellites are repetitive DNA sequences that are particularly vulnerable to replication errors without a functioning MMR system. Microsatellite instability (MSI) results from accumulated DNA replication mistakes and can be used as a surrogate marker for MMR gene mutations. Cancers that develop from germline MMR gene mutations usually exhibit MSI.

High levels of MSI (MSI-H) in tumor DNA can also result from a noninherited or sporadic epigenetic mechanism, causing somatic hypermethylation and subsequent transcriptional silencing of the MLH1 promoter. This epigenetic transcriptional silencing is typically associated with sporadic but not hereditary cancers (Beamer et al., 2012; Meyer, Broaddus, & Lu, 2009). Therefore, LS and MSI-H are not synonymous. In fact, 20% to 25% of all ECs show MSI, the majority of which (75%) are related to epigenetic promoter methylation (Longacre & Folkins, 2011). The majority of MMR gene mutations (90%) are associated with MLH1 and MSH2 genes, and in about 10% of the cases, PMS2 and MSH6 are mutated. Different gene defects confer various degrees of risk for cancer. Recent studies show that PMS1 and EPCAM also play an important role in LS and may represent a promising novel tool for the identification of LS patients. EPCAM is a gene located upstream of MSH2. Germline deletion of this gene may cause LS by epigenetic inactivation of the respective MSH2 allele (Kloor et al., 2011).

## Prognostic/Therapeutic Implications of MMR Abnormalities in EC

In contrast to the general consensus that individuals with MSI-H CRC tend to have a more favorable prognosis (Meyer, Broaddus, & Lu, 2009), the impact of MMR status on prognosis and/or therapy in EC is controversial. Some studies have noted that LS-associated ECs are often associated with adverse prognostic indicators, including nonendometrioid and undifferentiated histology, higher International Federation of Gynecology and Obstetrics (FIGO) grade, higher stage, and more frequent lymphovascular space invasion. Other studies failed to correlate prognostic information with MSI positivity. Larger studies with long-term clinical follow-up are required to definitively assess the impact of MMR genotype on therapy and outcome in EC patients (Meyer, Broaddus, & Lu, 2009).

## Screening/Diagnostic Criteria for Lynch Syndrome

Identification of LS probands in patients who initially presented with EC is believed to reduce the morbidity and mortality of metachronous CRC and other Lynch-related cancers in these patients and in their families, via effective screening and prevention strategies.

There is still no consensus on how to evaluate women with EC for LS. Clinical guidelines and criteria alone will miss a substantial number of women harboring MMR gene mutations, whereas universal testing with tumor studies or germline genetic testing in all women with EC will incur high costs. Tumor analysis followed by targeted genetic testing seems to be gaining favor as a cost-effective approach to identify potential LS in women presenting with EC.

**CLINICAL SCREENING CRITERIA**

The clinical screening tools for LS, which heavily rely on personal and family history, were initially established in 1991. The original Amsterdam criteria, known as the "3-2-1 rule," implied a diagnosis of LS if all of the following criteria were met: (1) ≥ 3 affected individuals with CRC, with 1 affected person being a first-degree relative (FDR) of the other 2; (2) CRC within 2 successive generations; and (3) 1 patient younger than 50. However, these criteria failed to include extracolonic malignancies such as EC, and the sensitivity and specificity were subsatisfactory (Chustecka, 2009; Resnick, Hampel, Fishel, & Cohn, 2009a). As our understanding of the clinical and histologic features of LS evolved, the Amsterdam criteria were revised in 1999 to include other noncolonic malignancies (Vasen, Watson, Mecklin, & Lynch, 1999). However, the revised version still has a heavy focus on colon cancer and fails to include women with sentinel gynecologic cancers and small family pedigrees. Hence its utility in the gynecologic oncology field is limited (Resnick et al., 2009a; 2009b).

The Bethesda criteria, created in 1997 and revised in 2002, had an important distinction from the Amsterdam criteria in that it not only outlined recommendations for identifying individuals with LS, but also described criteria for further MSI testing in CRC. To meet the Bethesda criteria, any one of the following must be true: (1) CRC diagnosed in a patient younger than 50; (2) the presence of synchronous, metachronous CRC, or other Lynch-associated tumors, regardless of age; (3) a CRC patient younger than 60 with MSI-H histology; (4) CRC diagnosed in one or more FDRs with a Lynch-related tumor, with one of the cancers being diagnosed before the age of 50; or (5) CRC diagnosed in two or more first- or second-degree relatives with Lynch-related tumors, regardless of age. The guidelines outlined the optimal approach to molecular evaluation of at-risk patients to include MSI or immunohistochemistry (IHC) analysis of tumors, followed by germline MSH2/MLH1 testing in patients with MSI-H tumors or tumors with a loss of expression of one of the MMR genes (Umar et al., 2004). The Bethesda guidelines, when compared with other existing Lynch clinical criteria, were found to be more sensitive for the identification of mutation carriers but with compromised specificity (Syngal, Fox, Eng, Kolodner, & Garber, 2000).

Both the Amsterdam criteria and Bethesda guidelines were historically established with a focus on screening CRC patients, and their performance characteristics have been well documented in the CRC population. However, studies have shown that solely using clinical guidelines/criteria could result in missing the diagnosis of many individuals with LS (Hampel et al., 2008).

**TISSUE MOLECULAR ANALYSIS**

MSI testing. Lynch syndrome can be tested for by identifying the presence of MSI in extracted tumor DNA using polymerase chain reaction (PCR) analysis. There is a panel of five National Cancer Institute–recommended microsatellite markers. By comparing the extent of microsatellite in DNA isolated from normal and tumor cells, the number of changed markers identified in tumors is generated, and results are interpreted as MSI-H (instability in two or more markers), MSI-low (MSI-L, only one of the five markers being unstable), and MS stable (no instability). It has been reported that EC patients with LS exhibit lower levels of MSI as compared to CRC patients. Thus, MSI testing in EC has the limitation of missing MSH6 mutations that may be MSI-L or MS stable (Hampel et al., 2006). In addition, MSI testing is unable to differentiate between MSI caused by epigenetic promoter methylation vs. germline mutations (Longacre & Folkins, 2011). More than 70% of ECs that are MSI-H may be due to MLH1 methylation.

IHC testing. The absence of one or more MMR proteins as a result of a mutation can be identified by IHC on fresh or archival tumor tissue, which can be technically done in most hospital pathology labs. When a panel of four proteins was tested (MLH1, PMS2, MSH2, and MSH6), IHC had a sensitivity of 91% and a specificity of 83% for detecting MSI-high in EC (Modica et al., 2007). Recently, a two-antibody panel (consisting of PMS2 and MSH6) has been found to be as effective as the four-antibody panel for detecting MMR abnormalities in CRC and gynecologic malignancies (Longacre & Folkins, 2011). Loss of MLH1 by IHC may also be caused by MLH1 promoter methylation rather than germline mutations, and a further MLH1 methylation assay should be performed to differentiate genetic (real LS) vs. epigenetic (sporadic EC) mechanisms (Longacre & Folkins, 2011; National Comprehensive Cancer Network [NCCN], 2012; Meyer et al., 2009; Umar et al., 2004).

Microsatellite instability analysis is more expensive and technically demanding than IHC. Immunohistochemistry has been shown to have a high concordance rate with MSI: 94% in both CRC and EC (Palomaki, McClain, Melillo, Hampel, & Thibodeau, 2009). The additional information provided by the staining pattern on IHC also can guide gene-specific DNA analysis. However, many favor combining MSI and IHC testing to minimize the likelihood of missing a diagnosis.

**Germline genetic testing.** Germline genetic testing for LS includes a combination of DNA sequencing and gene rearrangement analysis to identify mutation, deletion, duplication, and insertion within the MMR genes. It is considered a definitive/confirmatory test to establish LS diagnosis, but it is not an effective screening test (Longacre & Folkins, 2011). It should be utilized following IHC, MSI, and MLH1 methylation testing (Longacre & Folkins, 2011; NCCN, 2012; Umar et al., 2004), the results of which can guide targeted germline testing. Genetic testing of only the targeted gene will result in cost savings; if an MMR protein is missing per IHC, for instance, genetic testing for that particular gene can be initiated for a definitive diagnosis (Hampel et al., 2006). Once a specific mutation is identified in a woman with EC, her family members can undergo targeted predictive genetic testing for the same mutation.

**UNIVERSAL TUMOR ANALYSIS FOR CRC**

In 2009, the EGAPP Working Group at the Centers for Disease Control and Prevention published an evidence-based recommendation that all patients with a new diagnosis of CRC, regardless of age, ethnicity, or family history, should be screened for LS to identify opportunities to reduce morbidity and mortality in their families (EGAPP Working Group, 2009). Universal screening in CRC for LS has not only been found to be more sensitive than the revised Bethesda guideline, but also cost-effective, as it detects nearly twice as many cases of LS compared with targeting only younger patients. It has an incremental cost-effectiveness ratio comparable with that of other preventive services measured by quality-adjusted life-year gained (Mvundura, Grosse, Hampel, & Palomaki, 2010). Ladabaum et al. (2011) found that when all new tumors and three or more family members undergo genetic testing and surveillance guidelines are followed, cost is as little as $36,000 per life-year saved. This is well within the value of preventive health strategies. In particular, women with LS could improve their life expectancy by about 4 years if they have total abdominal hysterectomy (TAH) and bilateral salpingo-oophorectomy (BSO) and follow CRC surveillance guidelines (Ladabaum et al., 2011).

Many institutions have already implemented universal testing in CRC, and others are either interested or in the process of doing so. The American College of Surgeons Commission on Cancer (ACS/CoC) added a requirement for cancer registry abstraction of MSI test results on tumors from the colon, rectum, small intestine, and appendix (American Joint Committee on Cancer, 2011). While recognizing the trend in universal testing, current NCCN guidelines still refer to Amsterdam and Bethesda guidelines (NCCN, 2012).

Establishing a process for LS screening is complicated and has multiple patient and social implications. There are several key components to consider when planning a screening program. These include a process to obtain informed consent, cost of testing, patient access to genetic counseling, educational resources, genetic privacy, limited screening effectiveness, poor compliance, and psychosocial or emotional burdens to patients and their relatives. Each of these items can be a barrier to obtaining the desired outcome of the screening (Phillips, 2012; Hall, 2010). More evidence is needed to support the clinical and global benefits in order to justify a policy change toward population-based LS screening.

## Screening EC Patients for LS

Because the Amsterdam II criteria and Bethesda guidelines apply only to individuals with a diagnosis of CRC, their sensitivity is compromised in patients with small family pedigrees, families with a clustering of late-onset CRC, or those with a predominance of familial EC or other gynecologic tumors (Longacre & Folkins, 2011; Resnick et al., 2009a, 2009b; Ramsoekh et al., 2008). In a study examining 108 families that underwent molecular analysis for LS, 12 probands harbored MSH6 mutations, 7 of whom were diagnosed with EC as the primary cancer. The false-negative rate was 75% (9/12 patients) for the Amsterdam II criteria and 16.6% (false negative in 2/12) for the revised Bethesda guidelines (Ramsoekh et al., 2008). The high incidence of MSH6 mutation in families tested for LS made them less likely to fulfill clinical diagnostic criteria. Hampel et al. found that 8/13 (61.5%) EC patients with LS did not meet any published clinical criteria for LS and would have been missed if only personal and/or family histories were used for screening (Hampel et al., 2006).

Historically, the incidence of LS in women diagnosed with EC younger than 50 was thought to approach 10%. Microsatellite instability testing in EC patients younger than 50 has been proposed as a cutoff for screening. However, in two separate studies, the mean age at diagnosis of the probands was 54.1 and 54.8 years, unlike 48 years, which was reported in earlier studies (Resnick et al., 2009b). Limiting LS screening to patients with EC younger than 50 would result in missing MSH6 mutation and therefore leaving patients and families with LS undiagnosed.

Given the limitations of using personal/family cancer histories as well as pathologic risk factors, the need for a less restrictive and cost-effective algorithm for screening patients with EC for LS is apparent. As a step toward this goal, the Society of Gynecologic Oncologists (SGO) published guidelines in 2007 to assist triaging patients to a genetic counselor or genetic testing based on the level of perceived risk. The guidelines recommend genetic risk assessment for patients with a 20% to 25% chance of having LS. Genetic risk assessment is considered helpful for patients with a slightly lower risk (5%–10%; Lancaster et al., 2007). The NCCN guidelines also acknowledge that MSI testing for all CRC patients followed by MSH2 and MLH1 testing of MSI-H tumors) may be the most cost-effective approach for screening (NCCN, 2012).

The pendulum might be swinging toward screening strategies that encourage the identification of women who may benefit from hereditary cancer risk assessment and allow selection of patients who may not meet historic criteria but may warrant genetic screening. However, most of the available guidelines are still heavily based on an extensive family or personal history for determination of perceived risk or testing/screening. The NCCN guidelines, though acknowledging the emerging trend of universal screening of all CRC and EC regardless of age at diagnosis or family history, still recommend age < 50 as the testing criterion for EC patients (NCCN, 2012). In summary, less restrictive criteria for screening for LS in women with gynecologic cancers are needed, and their cost-effectiveness needs to be established.

## Evidence for Expanded Screening/Testing

A growing body of evidence shows that more generalized screening criteria are feasible and cost-effective to identify LS in women with EC. Tumor testing, rather than clinical history alone, has been incorporated into screening modalities for LS in EC patients. Hampel et al. compared the overall cost of IHC with that of MSI screening and found that IHC leads to fewer genes being sequenced (Hampel et al., 2008). In one study (Backes et al., 2009), IHC was performed on all EC tumors (140 cases) in one institution, > 90% of which were endometrioid adenocarcinoma. Loss of ≥ 1 MMR proteins was found in 30 patients (21%), including loss of both MLH1 and PMS2 in 24 patients and loss of both MSH2 and MSH6 in 4 patients. MSH6 loss was identified in two cases. None of the patients met the clinical criteria (Amsterdam II) for LS. This study highlights the limitation of using Amsterdam criteria for screening and also supports IHC testing of MMR proteins as a feasible and effective primary triage tool for LS evaluation in women with EC. Recommendations have been made to offer screening for LS among all women with newly diagnosed EC (Moline et al., 2013; Frei, 2011). Universal screening of all women with EC for LS will likely identify a greater number of mutation carriers; however, more data are needed to show that this strategy does not incur inappropriate costs to our health-care system.

Resnick et al. (2009b) compared the cost-effectiveness ratios and incremental cost-effectiveness ratios (ICERs) of four different strategies: (1) full-gene sequencing for women with EC who meet Amsterdam II criteria; (2) full-gene sequencing for women with EC; (3) full-gene sequencing for all women < 60 with EC; (4) IHC followed by single-gene sequencing for all women with EC. Incremental cost-effectiveness ratios, defined as the additional cost of a specific strategy divided by its additional health benefit, as compared with an alternate strategy, were determined for each strategy. A strategy that is more costly but more effective than an alternate strategy is considered cost-effective if its ICER is below $50,000 per life-year gained, a commonly used cutoff for cost-effectiveness analyses evaluating preventive health measures. Assuming that 40,000 new EC patients are diagnosed annually in the United States, prospective IHC evaluation of all tumor specimens followed by single-gene sequencing was found to be the most cost-effective strategy for detecting LS: 858 patients with LS were identified at a favorable ICER of $13,812. Full-gene sequencing for women with EC who met Amsterdam II criteria was the least costly but also detected the fewest number of patients (83).

Kwon et al. (2011) utilized the Markov chain Monte Carlo simulation model and examined the costs and benefits of six strategies to identify LS in a hypothetical cohort of women with EC. The six strategies were as follows: (1) Amsterdam II criteria; (2) < 50 years old with at least one FDR having a Lynch-associated cancer at any age; (3) IHC triage if age younger than 50; (4) IHC triage if age younger than 60; (5) IHC triage at any age if 1 FDR; and (6) IHC triage of all EC. For women with EC in the general population, the first two criteria were defined for direct genetic counseling and MMR gene sequencing, whereas the other four criteria were defined for IHC triage of EC followed by genetic counseling and testing if the IHC results were abnormal. Immunohistochemistry triage of women with EC having at least one FDR achieved an ICER of $9126 and will identify 755 cases (1.68%) of LS among 45,000 annual cases of EC, whereas universal IHC triage of all cases of EC identified most mutation carriers (827 cases of LS, 1.8%) and prevented the most CRC, albeit at a considerable increased cost ($648,494 per life-year gained). Furthermore, universal IHC triage for all EC patients is not practical, as it would require informed consent and discussion for all patients. The authors concluded that IHC triage of an EC tumor at any age with at least one FDR with a Lynch-associated cancer is a cost-effective strategy for detecting LS.

**LIMITATIONS**

Limitations inherent to these hypothetical simulation models must be considered. These include variability of LS prevalence within specific age subgroups, their CRC risks and mortality rates, and total lifetime costs for CRC treatment, the assumption that women in each strategy were matched for comorbidities and other Lynch-associated cancers, and an assumption of 100% compliance rate with CRC screening. Despite these limitations, Kwon’s study provided an estimate of the costs and benefits of various strategies to identify LS in a large cohort of women with EC, which would be difficult to achieve in a clinical setting. It is believed that the model used in this study should alert the clinicians to subject patients with EC having any FDRs with a LS-associated cancer to appropriate testing and genetic counseling. According to Dr. Lynch, a carefully taken history remains an important strategy in identifying patients who need a screening test (Barton, 2011). However, when family histories are unknown or incomplete or there are other reasons to justify a high level of suspicion, the clinician should individualize a strategy that may truly benefit the patient and not rigidly rely on hypothetical models for decision guidance (Kwon et al., 2011).

Despite their inherent limitations, these studies make good clinical and economic sense. They suggest population health benefits and feasibility that should move us closer to a national approach toward screening for LS in EC patients through multidisciplinary collaboration.

## Surveillance and Risk-Reducing Strategies for Gynecologic Cancers in LS

The cost-effectiveness of LS screening strategies relies on spreading the benefit across multiple family members (Ladabaum et al., 2011). Women with the diagnosis of EC and at least one FDR with LS-related cancer at any age should receive IHC triage (Kwon et al., 2011) and undergo further genetic counseling and testing if indicated. The actual uptake of genetic counseling and testing has been studied more extensively among patients identified with CRC pathologic screening as compared to EC. In CRC, the results ranged from 14% to 59% across studies. Impacting factors include cost (most of these studies offer free counseling), having children, family history, and social support (National Cancer Institute, 2012). In one study, based on family history and MMR IHC stains, 11% (15) of 140 EC cases were referred for genetic counseling. Only 3 out of the 15 (20%) had actually made the appointment. The acceptance of genetic counseling/testing is surprisingly low (Backes et al., 2009). Further investigation is needed to offer more insight into why individuals decide to undergo or decline genetic counseling and testing.

If individuals are identified as carriers for LS, given the substantial risks for CRC, frequent surveillance should be initiated, and patients should be counseled regarding available preventive measures. Prophylactic colectomy may be appropriately used in patients noncompliant for colonoscopy (Lynch & Casey, 2007).

Despite the paucity of evidence on effective screening methods for EC and OC, LS puts women at an increased lifetime risk for both gynecologic malignancies; based on expert consensus, screening is a reasonable option (Meyer et al., 2009; NCCN, 2012). The NCCN recommended that annual office endometrial sampling and/or transvaginal ultrasound may be judiciously applied at the clinician’s discretion. It is important to educate LS patients regarding the signs and symptoms of Lynch-related cancers (e.g., unusual vaginal bleeding, etc.). The effect of chemoprevention with oral contraceptives in the setting of LS is currently not known (NCCN, 2012).

Growing evidence shows a potentially life-saving benefit associated with prophylactic hysterectomy or TAH with BSO after age 35 or once child bearing is completed. These procedures have been demonstrated to be effective in reducing the risk of EC and OC in women who carry LS-related germline MMR mutations (Schmeler et al., 2006; NCCN, 2012). The surgery can also be timed with concurrent surgery for CRC. In Schmeler’s study, 315 women with documented germline LS-related mutations were identified. Of these 315 women, 61 had undergone prophylactic hysterectomy and 47 had undergone prophylactic BSO. They were matched with mutation-positive women who had not undergone the above-mentioned procedures. There were no recurrences of EC, OC, or primary peritoneal cancer in women who had undergone prophylactic surgery. One-third (33%) of the women in the control group developed EC and 5% of the women in the control group developed OC. In another study, women with LS could improve their life expectancy by about 4 years if they had TAH and BSO and followed CRC surveillance guidelines (Ladabaum et al., 2011).

Risk-reducing prophylactic surgery has also been shown to be comparatively less expensive than gynecologic surveillance in LS. These patients should be consulted for surgical complications, induction of surgical menopause, need for surgical staging if intraoperative evidence of malignancy is present, and the small likelihood of developing primary peritoneal carcinoma after TAH and BSO, as there have been such cases reported in LS patients (Lynch & Casey, 2007; Longacre & Folkins, 2011). Family members of LS patients should also undergo genetic testing to establish risk as well as surveillance for both EC/OC and CRC and counseling about prophylactic surgeries, as appropriate. A more thorough list of recommended screening and prevention options is available on the NCCN website (NCCN, 2012).

## Summary

Women with LS are at equal or higher risk for gynecologic cancers compared to their risk for CRC. Gynecologic cancer often precedes CRC as patients’ sentinel malignancy. Identifying these patients is believed to reduce their substantial risk for synchronous and metachronous tumors and has profound implications for reducing cancer-related morbidity and mortality in other family members. Current screening guidelines for identifying LS in women with EC vary but heavily rely on patient age and personal/family histories, with or without incorporation of tumor pathology. Because each of these strategies misses a significant proportion of women with LS, more inclusive screening strategies that make good economic and clinical sense are needed. In recent years, emerging evidence has shown that such strategies could be cost-effective, and implementation of such a strategy requires multidisciplinary collaboration and partnership. Advanced practitioners are well-situated to help identify patients at risk for LS at the patient level, by evaluating the family history and tumor characteristics and providing education about potential benefit to relatives, and at the system level, by adding their insights and knowledge to the discussion of universal screening.
